# Tackling inequalities in obesity: a protocol for a systematic review of the effectiveness of public health interventions at reducing socioeconomic inequalities in obesity among adults

**DOI:** 10.1186/2046-4053-2-27

**Published:** 2013-05-10

**Authors:** Clare L Bambra, Frances C Hillier, Helen J Moore, Joanne-Marie Cairns-Nagi, Carolyn D Summerbell

**Affiliations:** 1 Department of Geography, Wolfson Research Institute, Durham University Queen’s Campus, University Boulevard, Stockton-on-Tees TS17 6BH, UK; 2 School of Medicine, Pharmacy and Health, Wolfson Research Institute, Durham University Queen’s Campus, University Boulevard, Stockton-on-Tees TS17 6BH, UK

**Keywords:** Obesity, Socioeconomic inequalities, Public health, Adults

## Abstract

**Background:**

Socioeconomic inequalities in obesity and associated risk factors for obesity are widening throughout developed countries worldwide. Tackling obesity is high on the public health agenda both in the United Kingdom and internationally. However, what works in terms of interventions that are able to reduce inequalities in obesity is lacking.

**Methods/Design:**

The review will examine public health interventions at the individual, community and societal level that might reduce inequalities in obesity among adults aged 18 years and over, in any setting and in any country. The following electronic databases will be searched: MEDLINE, EMBASE, CINAHL, PsycINFO, Social Science Citation Index, ASSIA, IBSS, Sociological Abstracts, and the NHS Economic Evaluation Database. Database searches will be supplemented with website and gray literature searches. No studies will be excluded based on language, country or publication date. Randomized and non-randomized controlled trials, prospective and retrospective cohort studies (with/without control groups) and prospective repeat cross-sectional studies (with/without control groups) that have a primary outcome that is a proxy for body fatness and have examined differential effects with regard to socioeconomic status (education, income, occupation, social class, deprivation, poverty) or where the intervention has been targeted specifically at disadvantaged groups or deprived areas will be included. Study inclusion, data extraction and quality appraisal will be conducted by two reviewers. Meta-analysis and narrative synthesis will be conducted. The main analysis will examine the effects of 1) individual, 2) community and 3) societal level public health interventions on socioeconomic inequalities in adult obesity. Interventions will be characterized by their level of action and their approach to tackling inequalities. Contextual information on how such public health interventions are organized, implemented and delivered will also be examined.

**Discussion:**

The review will provide evidence, and reveal any gaps in the evidence base, of public health strategies which reduce and prevent inequalities in the prevalence of obesity in adults and provide information on the organization, implementation and delivery of such interventions.

**Trial registration:**

PROSPERO registration number: CRD42013003612

## Background

There is a strong and growing evidence base that shows the impact of obesity on short and long-term functioning, health and wellbeing [1]. Obesity has been linked to chronic diseases such as diabetes, coronary heart disease, stroke, hypertension, osteoarthritis and certain forms of cancer [2] thus demonstrating the significance of obesity for health and wellbeing. Internationally, adult obesity rates continue to rise as seen in the latest Organisation for Economic Co-operation and Development (OECD) figures. According to these figures, more than half (50.3%) of the OECD member countries have an adult population who were classified as obese or overweight [3]. This staggering figure shows that adult obesity rates have doubled, and in some cases tripled, since 1980. It has been estimated that based on healthcare costs in the United Kingdom (UK) the burden of diseases associated with obesity will cost approximately £10 billion per year by 2050 [4]. Consequently, the number of early deaths, long-term incapacity and reduction in quality of life will escalate. Taking England as an example, recent figures show that just over a quarter of adults (26% of both men and women over the age of 16) were classified as obese [5]. Therefore, tackling obesity is rightly highlighted as one of the major contemporary public health concerns and vital for addressing socioeconomic inequalities in poor health.

### Inequalities in obesity

Studies have found that in most European countries, the United States and Australia socioeconomic inequalities in obesity and associated risk factors for obesity are widening [1,3,4,6,7]. A global assessment of these socioeconomic inequalities in obesity is important in order to prevent and reduce the development of chronic diseases.

In the UK, as is the case in most other high income countries, obesity is more prevalent in the lowest income quintile [8]. There is little evidence of change over time [9]. Mackenbach and colleagues’ (2008) research shows that socioeconomic inequalities in obesity are larger among women, particularly in the southern region of Europe, whereby in some countries education-related inequalities in obesity are almost four times higher for those with the least education [10]. Moreover, it has been found that there is also a clear socioeconomic gradient in body mass index (BMI) for residents living in more deprived neighborhoods, independent of individual socioeconomic position [11]. Geographical inequalities are also evident in England, with hotspots in the North East, Yorkshire and Humber, and the East and West Midlands [9] and a clear north–south divide [5]. Longitudinal analyses also suggest that the social gradient in obesity is associated with the accumulation of disadvantage throughout the life course contributed to widening inequalities in obesity in adulthood and this trend is more pronounced for women [8].

### Policy context

While tackling obesity is high on the public health agenda both in the UK and internationally there is a lack of accessible information to inform policymakers and service commissioners of services about what types of interventions are most effective in reducing such inequalities in obesity. The Priority Public Health Conditions group (Task Group 8) of the Department of Health commissioned Strategic Review of Health Inequalities in England post-2010 (Marmot Review) [12,13] noted this evidence gap as did the EPPI Centre report on obesity (2008), which called for future systematic reviews to examine the effectiveness of interventions in reducing inequalities [14].

Internationally, there have been similar calls for research in this area. Robertson *et al.* identify the need for ‘*evidence of the reach and penetration of interventions in lower income groups’* as a priority for research [7]. The World Health Organization (WHO) Commission on the Social Determinants of Health also highlighted the relative importance attached to *‘the development and testing of social determinants of health indicators and intervention impact evaluation’*[15]. Further, there is increasing recognition among policymakers that in order to effectively tackle complex health problems, such as obesity, and to reduce health inequalities, policy action across different intervention levels (including individual, community and society) as well as across the life course (childhood to adulthood) is required. Therefore, it is critical that evidence on the effectiveness of different types of interventions that tackle inequalities in obesity at these various levels are systematically identified, appraised and synthesized. To our knowledge there are no systematic reviews to date that have examined the effectiveness of interventions to reduce socioeconomic inequalities in obesity internationally.

The systematic review proposed here will address the aforementioned knowledge gap by reviewing primary studies that have considered the effectiveness of interventions that seek to reduce socioeconomic inequalities in obesity among adults. We approach this review in a whole systems way incorporating a multilevel perspective. This review will subsequently examine public health interventions at the individual, community and societal level [4,8] that tackle inequalities in obesity. It will also examine organization, implementation and delivery of such interventions. This adult review complements our previous systematic review project of interventions to reduce inequalities in childhood obesity [16].

### Tackling inequalities in obesity using a whole systems approach: developing an intervention framework

The Foresight review of obesity stressed the importance of a whole systems approach to tackling the ‘obesity epidemic’ [4]. Within this approach interventions are needed that target the wider social determinants of obesity [6]. Whitehead and Dahlgren’s [17] oft-cited social model of health rainbow considers various levels of influence on health: individual (including biological and lifestyle factors), socio-cultural, living and working conditions and broader socioeconomic, cultural and environmental conditions. These are potential levels for intervention for reducing obesity. Using a whole systems approach and considering these various levels of intervention, we have developed an intervention framework (Table 1), which was used in our previous protocol for child obesity; however, we have updated it to reflect our focus on adult obesity in this protocol.

**Table 1:** A framework for tackling inequalities in obesity

**APPROACH TO TACKLING HEALTH INEQUALTY**		**LEVEL OF INTERVENTION**			
		**Individual**	**Community**	**Societal**	
		***Strengthening individuals***	***Strengthening communities***	***Improving living and work environment***	***Promoting healthy macro policies***
**Disadvantage Gap**	*Targeted*	Health education, health promotion and social marketing; Diet and exercise advice and counseling; Weight management advice and monitoring; Conditional cash transfers; Lifestyle counseling; Exercise on prescription.	Community health and fitness centers; Health trainers; Group, work or community based exercise programs; Group, work or community diet, lifestyle, or weight management advice and/or counseling; Healthy eating campaigns in workplaces; Group or community organized education or support; Localized point of sale social marketing; Neighborhood based physical activity programs.		

**Gradient**	*Universal*			Access to physical fitness facilities (e.g. gym subsidies); Availability of healthy food; Green spaces, walk-ability and the built environment; Traffic light labeling.	Restrictions on advertising high fat and high sugar foods; Food prices and agricultural subsidies (e.g. changing the Common Agricultural Policy); Fiscal measures to regulate supply and demand (e.g. taxing high fat and high sugar foods).

An overwhelming number of studies have focused on interventions at the individual level including educational, behavioral and pharmaceutical approaches. Swinburn *et al.*[18] argue that these types of interventions have only had a modest impact on reducing obesity and that there is a real need to focus on environments that are either ‘obesogenic’ (promotes obesity) or ‘lepogenic’ (promote leanness). They classify the environment into micro- and macro-environments. Micro-environments include how individuals interact with schools, workplaces, homes, and neighborhoods. Macro-environments include education, health systems, government, the food industry and societal attitudes and beliefs. Within these environments we may categorize them into physical, economic, political, or sociocultural.

The intervention framework proposed in this review considers these broader determinants of health in order to understand the mechanisms through which the different layers of intervention may operate to reduce socioeconomic inequalities in health. There are four levels of interventions to tackle inequalities: 1) strengthening individuals (person based strategies to improve the health of disadvantaged individuals); 2) strengthening communities (improving the health of disadvantaged communities and local areas by building social cohesion and mutual support); 3) improving living and school environments (reducing exposure to health-damaging material and psychosocial environments across the whole population); and 4) promoting healthy macro policy (improving the macro-economic, cultural and environmental context which influence the standard of living achieved by the whole population). According to Graham and Kelly, these interventions are underpinned by one of three different approaches to health inequality: 1) disadvantage (improving the absolute position of the most disadvantaged individuals and groups); 2) gap (reducing the relative gap between the best and worst off groups); or, 3) gradient (reducing the entire social gradient) [19]. Interventions are thus either targeted (such as individual level interventions underpinned by health as disadvantage) or universal (such as living and working conditions interventions that potentially influence the entire social gradient in health). In the proposed systematic review, the obesity interventions will be grouped according to this framework.

## Methods/Design

The review will follow the same procedure carried out for the systematic review of the effectiveness of public health interventions at reducing socioeconomic inequalities in obesity among children [16]. The review will be carried out following established criteria for the good conduct and reporting of systematic reviews [20,21]. A Study Steering Group comprising key stakeholders from the UK policy and research communities, international representatives, a statistician and a health economist will guide the research. The review is registered with the PROSPERO International Prospective Register of Systematic Reviews (registration number: CRD42013003612).

### Objectives

This project has two objectives:

1. To systematically review the effectiveness of public health interventions (individual, community and societal) in reducing socioeconomic inequalities in obesity among adults.

2. To establish how such public health interventions are organized, implemented and delivered.

### Interventions

The review will examine public health interventions at the individual, community and societal level that might reduce inequalities in obesity among adults aged 18 years and over, in any setting, in any country. The review will utilize the intervention framework (Table 1) and group interventions as individual, community or societal with acknowledgement that some interventions might be cross-cutting. For example an individual level intervention could be a relatively complex weight management program providing dietary and physical activity education with behavioral counseling delivered individually (as opposed to group-based) or could simply be a gift card given to low-income families for the purchase of fruits and vegetables. An example of a community level intervention may be access to a community fitness center (with/without the addition of physical activity education), or a workplace health promotion campaign involving dietary and physical activity education. A societal level intervention may also be a workplace health promotion campaign but one that involves an environmental change such as healthy food choices in the work canteen. The review will consider public health strategies which might reduce existing inequalities in the prevalence of obesity as well as those interventions which might prevent the development of inequalities in obesity. However, clinical interventions such as those using drugs or surgery and laboratory-based studies will be excluded. We will also exclude studies of interventions designed for adults with a critical illness or severe comorbidities (such as diabetes, metabolic syndrome, hypertension and mental health disorders).

### Study designs

A rigorous and inclusive international literature search will be conducted for all randomized and nonrandomized controlled trials, prospective and retrospective cohort studies (with/without control groups) and prospective repeat cross-sectional studies (with/without control groups) of the effectiveness of public health interventions at reducing inequalities in childhood obesity. Studies with a duration of at least 12 weeks (combination of intervention and follow up) will be included; an inclusion criterion used in previous Cochrane reviews of interventions aimed at preventing obesity in children [22] and of the effectiveness of exercise for weight loss in adults with overweight or obesity [23].

### Search strategy

The search strategy (Additional file 1) will include the following electronic database searches (host sites given in parentheses) MEDLINE (Ovid), EMBASE (Ovid), CINAHL (EbscoHost), PsycINFO (EbscoHost), Social Science Citation Index (Web of Science), ASSIA (CSA), IBSS (EBSCO), Sociological Abstracts (CSA), and the NHS Economic Evaluation Database (NHS CRD). The skills of a trained information scientist (HJM) will be used to develop and implement the electronic searches. All databases will be searched from start date to present. We will not exclude papers on the basis of language, country or publication date.

We will supplement the electronic database searches with website and gray literature searches. We will hand search the bibliographies of all included studies and request relevant information on unpublished and in-progress research from key experts in the field. In addition, we will hand search the last two years of the most common five journals revealed by the electronic searches as well as journals identified by experts in the subject area.

### Outcomes

In terms of outcomes, we will only include studies if they include a primary outcome that is a proxy for body fatness (weight and height, body mass index, waist measurement/waist to hip proportion, percentage fat content, skin fold thickness, and ponderal index in relation to childhood obesity). Data on related secondary outcomes (such as physical activity levels; dietary intake; blood results, such as cholesterol and glucose levels) will also be extracted from those studies which have a primary outcome. We will include both measured and self-reported outcomes. Studies will only be included if they have examined differential effects with regard to socioeconomic status (education, income, occupation, social class, deprivation, and poverty) or the intervention must have been targeted specifically at disadvantaged groups (for example, unemployed, low SES and low income) or deprived areas. Data on the organization, implementation and delivery of interventions will be extracted using existing methodological tools which assess the implementation of complex public health interventions [24], adapted and refined for the purposes of this review. Examples of the implementation components that will be examined include: theoretical underpinning; implementation context; experience of intervention team (planners and implementers); consultation/collaboration processes (planning and delivery stages); and resources (for example, time, money, staff, and equipment).

### Data extraction and quality appraisal

The initial screening of titles and abstracts and full paper inclusion will be conducted by one reviewer (FCH or JMC) with a random 10% of the sample checked by a second reviewer (HJM or FCH). Data extraction and methodological quality appraisal of the included studies will be conducted by one reviewer (FCH, JMC, CLB, or CDS) using established data extraction forms [20,25-30] and will be checked by a second reviewer (FCH, JMC, CLB, or CDS). Any discrepancies will be resolved through discussion between the authors and, if consensus is not reached, with the project lead (CLB). The methodological quality of the included studies will also be appraised using the Cochrane Public Health Review Group recommended Effective Public Health Practice Project Quality Assessment Tool for Quantitative Studies [31], which includes, among other things, an examination of sampling strategy, response and follow-up rates, intervention integrity, statistical analyses and assessment of adjustment for confounders. We will use the quality appraisal criteria for descriptive purposes and to highlight variations between studies.

### Analysis and synthesis

Where possible, meta-analysis will be used to synthesize data using Comprehensive Meta-Analysis (Biostat, Englewood, NJ, USA) based on the primary outcomes. A fixed effect model will be used for the meta-analysis, unless there is evidence of heterogeneity between studies, in which case a random effect model will be used. The presence of heterogeneity will be investigated with the use of likelihood ratio test statistic, while funnel plot will be considered to explore publication bias. However, where meta-analysis is not possible, narrative synthesis will be conducted. We will report our analyses in accordance with PRISMA guidelines [32]. The main analysis will examine the effects of 1) individual, 2) community and 3) societal level public health interventions on socioeconomic inequalities in obesity, using the multi-dimensional framework outlined in Table 1. Where data permits, we will conduct demographic subgroup analysis by age, gender and ethnicity.

## Discussion

The review will consider public health strategies that reduce existing inequalities in the prevalence of obesity as well as those interventions that might prevent the development of inequalities in obesity. The review will also serve as a mapping exercise of the types of interventions that have been evaluated in relation to tackling inequalities in obesity in adults, thereby highlighting any gaps in the evidence base. The review will also seek to establish how public health interventions that might reduce or prevent inequalities in obesity are organized, implemented and delivered. Context is increasingly recognized as an important factor in the success of public health interventions [33]. However, the assessment of implementation has not really featured strongly in previous obesity reviews. We will therefore develop, refine and apply existing methodological tools that assess the implementation of complex public health interventions [24].

We anticipate that a sizeable amount of literature will be located for synthesis as a result of our extensive search strategy, along with gray literature and website searches, and inclusive study design criteria. The size of the available evidence base will also be extended because we are looking at different levels of intervention: individual, community and societal, and we will evaluate interventions that are targeted at deprived groups or areas as well as those that include comparative data on the effects of interventions on differential impacts across two or more socioeconomic groups. The study design inclusion criteria in the review are broad given that while trials of individual-, and even community-level interventions are likely, we expect a dearth of experimental studies in relation to societal-level interventions as societal-level interventions tend not to be easily evaluated using experimental study designs [8]. Further, other recent systematic reviews of the effects of societal level public health interventions on socioeconomic inequalities in health have located few relevant experimental studies [34]. Full papers of all studies that fit our population, intervention, design and health outcome inclusion criteria will be examined, even if there is no mention of socioeconomic inequalities in the abstract; in this manner we will reduce the possibility of excluding studies that undertook subgroup analyses by socioeconomic status but did not publish the findings in the abstract. These strategies will increase the comprehensiveness of the search strategy and, therefore, the quality of the final synthesis.

We plan to hold an ‘implications for policy and practice’ review dissemination workshop with invited NHS commissioners with responsibilities for obesity; Department of Health policy makers with responsibility for obesity and inequalities; user group representatives (for example, community groups, schools and employer organizations, trade union congress); along with UK research network representatives (for example, Faculty of Public Health, Nutrition Society, UKPHA) to discuss the results, aid write up and facilitate translation of the findings into practice. Once the technical report and executive summary are finalized a short ‘key findings’ summary of the research will be sent to relevant stakeholders. The research will be disseminated via national and international academic/practitioner cross-over conferences, and a policy orientated summary paper will be published on an open access basis so that it is freely available to practitioners and the public.

## Competing interests

The authors declare that they have no competing interests.

## Authors’ contributions

CLB is Principal Investigator and led the writing of the manuscript. FCH is project manager and co-investigator and contributed to the writing and revision of the manuscript. HJM, JMC and CDS are co-investigators and contributed to the writing and revision of the manuscript. All authors read and approved the final manuscript.

## Supplementary Material

Additional file 1 Search Strategy – MEDLINE (Ovid) 1946 to 10 October 2012. Click here for file

## References

[B1] World Health Organisation Obesity: preventing and managing a global epidemic: Report of a WHO consultation of Obesity 2000 Geneva: World Health Organisation Technical Report Series 894 11234459

[B2] Cross-Government Obesity Unit Department of Health, Department of Children Schools and Families: Healthy Weight, Healthy Lives: A Cross-Government Strategy for England 2008 London: The Stationary Office

[B3] OECD Obesity and the Economics of Prevention: Fit not Fat 2010 Paris: OECD Publishing

[B4] Butland B Jebb S Kopelman P McPherson K, Thomas S, Mardell J, Parry V: Tackling obesities: Future choices - Project Report 2007 London: Government Office for Science

[B5] Health and Social Care Information Centre Statistics on obesity, physical activity and diet: England, February 2009 2009 Leeds

[B6] Friel S Chopra M Satcher D Unequal weight: equity oriented policy responses to the global obesity epidemic BMJ 2007 335 1241 1243 10.1136/bmj.39377.622882.47 18079548 PMC2137064

[B7] Robertson A Lobstein T Knai C Obesity and socio-economic groups in Europe: Evidence review and implications for action. Report SANCO/2005/C4-NUTRITION-03 2007 Brussels: European Commission

[B8] Law C Power C Graham H Merrick D Obesity and health inequalities Obes Rev 2007 8 19 22 17316296 10.1111/j.1467-789X.2007.00312.x

[B9] White M Adamson A Chadwick T Dezateux C Griffiths L Howel D Kelly S Law C Li L Lo Conte R Power C Stamp E The changing social patterning of obesity: an analysis to inform practice and policy development. Final report to the Policy Research Programme 2007 London: Department of Health

[B10] Mackenbach JP Stirbu I Roskam A-JR Schaap MM Menvielle G Leinsalu M Kunst AE Socioeconomic inequalities in health in 22 European countries N Engl J Med 2008 358 2468 2481 10.1056/NEJMsa0707519 18525043

[B11] van Lenthe FJ Mackenbach JP Neighbourhood deprivation and overweight: the GLOBE study Int J Obes 2002 26 234 240 10.1038/sj.ijo.0801841 11850756

[B12] Bambra C Joyce K Maryon-Davies A Strategic Review of Health Inequalities in England Post-2010 (Marmot Review) (Task Group 8: Priority public health conditions: final report) Book Strategic Review of Health Inequalities in England Post-2010 (Marmot Review) (Task Group 8: Priority public health conditions: final report 2009 London: University College London

[B13] Bambra C Joyce KE Bellis MA Greatley A Greengross S Hughes S Lincoln P Lobstein T Naylor C Salay R Wiseman M Maryon-Davis A Reducing health inequalities in priority public health conditions: using rapid review to develop proposals for evidence-based policy J Public Health 2010 32 496 505 10.1093/pubmed/fdq028 20435581

[B14] Woodman J Lorenc T Harden A Oakley A Social and environmental interventions to reduce childhood obesity: a systematic map of reviews. EPPI-Centre Report No. 1610 2008 London: EPPI-Centre, Social Science Research Unit, Institute of Education, University of London

[B15] WHO Commission on the Social Determinants of Health Closing the gap in a generation: health equity through action on the social determinants of health. Final Report of the Commission on Social Determinants of Health 2008 Geneva: World Health Organisation

[B16] Bambra C Hillier F Moore H Summerbell C Tackling inequalities in obesity: a protocol for a systematic review of the effectiveness of public health interventions at reducing socioeconomic inequalities in obesity amongst children Syst Rev 2012 1 16 22587775 10.1186/2046-4053-1-16 PMC3351709

[B17] Whitehead M Dahlgren G What can be done about inequalities in health? Lancet 1991 338 1059 1063 10.1016/0140-6736(91)91911-D 1681366

[B18] Swinburn B Egger G Raza F Dissecting obesogenic environments: the development and application of a framework for identifying and prioritizing environmental interventions for obesity Prev Med 1999 29 563 570 10.1006/pmed.1999.0585 10600438

[B19] Graham H Kelly M Health inequalities: concepts, frameworks and policy 2004 London: Health Development Agency

[B20] Centre for Reviews and Dissemination Undertaking systematic reviews of research on effectiveness. CRD’s guidance for carrying out or commissioning reviews 2nd edition 2001 York: University of York

[B21] Higgins J Green S Cochrane Handbook for Systematic Reviews of Interventions Version 5.1.0 [updated March 2011] 2011 The Cochrane Collaboration Available from http://www.cochrane-handbook.org

[B22] Summerbell C Waters E Edmunds L Kelly S Brown T Campbell K Interventions for preventing obesity in children Cochrane Database Syst Rev 2008 3 CD001871 10.1002/14651858.CD001871.pub2 16034868

[B23] Shaw K Gennat H O'Rourke P Del Mar C Exercise for overweight or obesity Cochrane Database Syst Rev 2006 4 CD003817 17054187 10.1002/14651858.CD003817.pub3 PMC9017288

[B24] Egan M Bambra C Petticrew M Whitehead M Reviewing evidence on complex social interventions: appraising implementation in systematic reviews of the health effects of organisational-level workplace interventions J Epidemiol Community Health 2009 63 4 11 18718981 10.1136/jech.2007.071233 PMC2596297

[B25] Bambra C Egan M Thomas S Petticrew M Whitehead M The psychosocial and health effects of workplace reorganisation. 2. A systematic review of task restructuring interventions J Epidemiol Community Health 2007 61 1028 1037 10.1136/jech.2006.054999 18000123 PMC2465678

[B26] Bambra C Whitehead M Sowden A Akers J Petticrew M A hard day’s night? The effects of Compressed Working Week interventions on the health and work-life balance of shift workers: a systematic review J Epidemiol Community Health 2008 62 764 777 10.1136/jech.2007.067249 18701725

[B27] Bambra CL Whitehead MM Sowden AJ Akers J Petticrew MP Shifting Schedules: the health effects of reorganizing shift work Am J Prev Med 2008 34 427 434 10.1016/j.amepre.2007.12.023 18407011

[B28] Deeks J Dinnes J D’Amico R Sowden A Sakarovitch C Song F Petticrew M Altman D Evaluating non-randomised intervention studies Health Technol Assess 2003 7 1 173 14499048 10.3310/hta7270

[B29] Egan M Bambra C Thomas S Petticrew M Whitehead M Thomson H The psychosocial and health effects of workplace reorganisation 1: a systematic review of interventions that aim to increase employee participation or control J Epidemiol Community Health 2007 61 945 954 10.1136/jech.2006.054965 17933951 PMC2465601

[B30] Joyce K Pabayo R Critchley J Bambra C Flexible working conditions and their effects on employee health and wellbeing Cochrane Database Syst Rev 2010 2 CD008009 20166100 10.1002/14651858.CD008009.pub2 PMC7175959

[B31] Effective Public Health Practice Project Quality Assessment Tool for Quantitative Studieshttp://www.ephpp.ca/tools.html

[B32] Liberati A Altman DG Tetzlaff J Mulrow C Gøtzsche PC Ioannidis JP Clarke M Devereaux PJ Kleijnen J Moher D The PRISMA statement for reporting systematic reviews and meta-analyses of studies that evaluate healthcare interventions: explanation and elaboration BMJ 2009 339 b2700 10.1136/bmj.b2700 19622552 PMC2714672

[B33] Chow CK Lock K Teo K Subramanian S McKee M Yusuf S Environmental and societal influences acting on cardiovascular risk factors and disease at a population level: a review Int J Epidemiol 2009 38 1580 1594 10.1093/ije/dyn258 19261658 PMC2786248

[B34] Bambra C Gibson M Sowden A Wright K Whitehead M Petticrew M Tackling the wider social determinants of health and health inequalities: evidence from systematic reviews J Epidemiol Community Health 2010 64 284 291 10.1136/jech.2008.082743 19692738 PMC2921286

